# Practical Results from Examination of the Stomach Contents

**Published:** 1902-03

**Authors:** E. Guy Hopkins

**Affiliations:** Richmond, Va.


### PRACTICAL RESULTS FROM EXAMINATION OF THE STOMACH CONTENTS.*

BY E. GUY HOPKINS, M.D.,

RICHMOND, VA.

Americans have been called a nation of dyspeptics, and, while the statement may be an exaggeration, the fact remains that gastric disorders constitute a large proportion of the average doctor's practice. The frequency of the trouble and the unsatisfactory results that follow its empirical treatment have impressed me with

*Read before the Richmond Academy of Medicine and Surgery.

the importance of making a more accurate diagnosis, and impelled me to study the recent methods practiced in the clinical laboratory by which the essential cause of the frequently complex group of symptoms can be isolated. In every disease the early and correct diagnosis must precede the logical and successful treatment. The frequently recognized difference of ability on the part of members of the medical profession is usually a difference as a diagnostician, not as a therapeutist.

No one who claims to be progressive can afford to be ignorant of the results which often follow the chemical and microscopical examination of the stomach contents; and no one who wishes to be successful can afford not to avail himself of the indications they reveal for direct and appropriate dietetic and medicinal treatment. No one would treat a case of Bright's disease without examining the urine; yet many still prescribe for a case of dyspepsia without examining the stomach contents. In reality, the work of Boas, Kussmaul, and others in gastric pathology has developed diagnostic points of greater importance than those brought out by Bright and Christison in renal pathology, because of the greater frequency of diseases of the stomach, as compared to diseases of the kidney, and their greater amenability to treatment.

The examination of the contents of the stomach is often the only means of arriving at a definite diagnosis; and it affords an immediate and accurate insight into the actual pathological processes present. By it we may determine in what way the functions of the stomach are perverted—whether the motor or secretory functions are increased, diminished or absent.

The peristaltic action of the stomach may be increased, the therapeutic indication would be sedative; it may be diminished, the treatment indicated stimulative. Lactic acid may be found present after a test meal free from lactates, a condition almost pathognomic of cancer, making it necessary to consider the advisability of an exploratory incision. Gases, such as sulphuretted hydrogen, carbon dioxid, etc., may be found, indicating fermentation and calling for sterile food and gastro-intestinal antiseptics. Bile may be persistently present, indicating stenosis of the descending part of the duodenum. Bacteria are always present, but certain types have special significance. Abundant sarcinas, when present, contraindicate the existence of carcinoma, but point to benign stenosis or greatly diminished motor function. The Oppler-Boas bacillus is found in the majority of cases of cancer, rarely in other affections. Mucus, in large quantity, indicates a chronic gastritis. Fragments of tissue found in the wash-water are of great diagnostic value in showing the real state of the gastric mucosa; and their microscopical examination often differentiates gastritis from a neurosis, or determines the presence of malignant growth.

As illustrative of the practical results of the examination of the stomach contents, I have selected five cases to report, from twenty-six which I have recently had under observation. These five are taken because they are all chronic cases that had been treated by the usual routine methods without success, and also because they represent a variety of conditions of gastric derangement which they typify. All yielded readily to the treatment indicated by the analyses except Case II, which was one of inoperable carcinoma of the cardia. In regard to the technique adopted in these examinations, it is merely necessary to say that the test meal of Ewald, consisting of a roll, or two pieces of toast, and a glass-and-a-half of water, was generally chosen because of its convenience; that of Boas being used only in cases of suspected carcinoma. Dimethylamidoazobenzol and phloroglucin-vanillin solutions were the tests for free hydrochloric acid. Ufflemann's and Strauss's tests were used for lactic acid, and Toppfer's method was adopted in the quantitative estimation of the factors of acidity.

CASE I. *Carcinoma of the Pylorus.* Capt. W., aged 44 years; patient of Dr. Tayloe, of North Carolina; admitted to St. Luke's Hospital Oct. 23, 1900; family healthy. Had been suffering from indigestion, which had gradually grown worse, for eighteen months. Food was retained in the stomach for an hour or two with great discomfort, then vomited. There was no pain, hematemesis or cachexia; a tumor could be felt in the upper part of the abdomen; he had gradually lost forty pounds in weight. Examination of the stomach contents, after a test meal, showed complete absence of free hydrochloric acid and the presence of about two-tenths per cent. lactic acid. A diagnosis of cancer of the pylorus was made, and pylorectomy was performed by Dr. Stuart McGuire, October 30, 1900. Microscopical examination of the specimen removed verified the chemical diagnosis. The patient, when last heard from, December 15, 1900, had gained thirty-eight pounds in weight, and was feeling perfectly well.

CASE II. *Carcinoma of the Cardiac End of the Stomach.* Mr. F., patient of Dr. Hodges, had a history of injury to the back, followed by peculiar nervous symptoms and pain in the back, chest and limbs, which the patient was forced to control by alcohol and morphine. Swallowing gradually became so difficult that he had to confine himself to liquid food. The diagnosis lay between nervous spasm of the cardia, benign stenosis and carcinoma. Upon the introduction of the stomach tube after the ingestion of some water, a small quantity of bloody fluid and fragments of tissue, which had been dislodged by the tube, were expelled. Microscopic examination revealed the structure of carcinoma. This case, on furthur examination, was deemed inoperable, and palliative treatment was instituted.

CASE III. *Achylia Gastrica.* Mrs. J., apparent age 45, had suffered for over a year with gastric discomfort, eructations of gas after eating, alternating diarrhea and constipation, heavy bearing down feeling in the abdomen, flatulence and discharge of yellow mucus and frothy material at stool. She had become very much weakened; was frequently disable for household duties; and had been treated with every known anti-ferment, tonic and digestant, but without success. An examination of the gastric contents, made March 9, 1901, showed an entire absence of free and combined hydrochloric acid, and a total acidity of 16, due to acid salts and volatile acids. The microscope showed abundant bacteria and yeast. The line of treatment adopted was to supply the deficient gastric secretion, to prevent fermentation, and to furnish a diet as easily digested under the existing conditions as possible. The first indication was met by giving dilute hydrochloric acid, twenty drops fifteen minutes after eating, and the same quantity half an hour later; the second by a mixture of resorcin, salicylate of bismuth and powdered rhubarb; and the third, by a soft, well-cooked finely divided, mixed diet, free from fats and sweets. Since instituting this treatment, the patient has steadily improved. She is stronger and able to attend to her work, has gained weight, the gastric distress and abdominal pain have disappeared, the bowels have become regular, and she is rarely troubled with excessive flatulence.

CASE IV. *Hyperchlorhydra.* Mrs. T., aged forty years, patient of Dr. Stuart McGuire, for three years has suffered from indigestion and symptoms of lithemia, viz.: headache, malaise, vertigo, constipation, etc.; and for a long time had confined herself to the Haig dietary, but without relief. She was first seen by the writer February 20, 1901. Her condition was markedly neurasthenic; she complained of intense discomfort in the stomach, somewhat relieved immediately after eating, but worse after an hour or two; acid eructations; utter wretchedness; inability for mental or physical activity, and a peculiar feeling of constriction at the top of the head. Examination of the stomach contents showed free hydrochloric acid 70, or nearly twice the normal, and total acidity, 100. The line of treatment adopted was directed to sedation of the nervous system and neutralization of the excessive acidity of the gastric juice. The first was accomplished by rest, static electricity, tincture of valerian and Hoffman's anodyne. The second indication was met by a diet consisting of a maximum of easily digested food, a minimum of carbohydrate food, and direct neutralization of the acid by the alkalis, sodium bicarbonate and calcined magnesia. Gastric discomfort was immediately relieved, and the neurasthenic condition gradually yielded to sedative treatment, so that now she feels pefectly well and has stopped all medicine, but still keeps up the prescribed diet.

CASE. V. *Atonic Dilatation with Subacidity.* Mrs. W., aged forty-five, had suffered from dyspeptic symptoms and headache for years. These had been attributed to chronic lithemia, or derangement of the liver. Her dyspeptic symptoms had gradually grown worse, and symptoms of serious intestinal disorder made their appearance. On December 3, 1900, when the writer first saw her, she had just returned from a New York sanitarium where a diagnosis of intestinal tuberculosis had been made, and the case regarded as hopeless. She was greatly emaciated, weighed seventy pounds; her jaws and limbs were frequently in that peculiar state of tonic contraction known as tetany; and she was so weak that she could not raise herself in bed without assistance; the heart action was weak and irregular; appetite completely lost; and nausea constant and attacks of vomiting frequent. Diarrhea was excessive, the stools being very offensive, light-colored, frothy and mixed with mucus; flatulence was also excessive. Although apparently contraindicated, analysis of the stomach contents was thought advisable. This was made December 5. Free hydrochloric acid was absent; combined hydrochloric acid, 4; and volatile organic acids, but no lactic, were present. In subsequent washing of the stomach it was found that it invariably contained food ingested the day before, and that its capacity was considerably above the normal. The treatment was first directed to controlling the tetany, for which bromides and morphine were necessary. Dilute hydrochloric acid was given, to supply the deficiency in the gastric juice and for its antiseptic action, in doses of twelve drops, gradually increasing to twenty drops, after eating. Tincture of digitalis, fifteen drops,[1] with whiskey, a half-ounce, was given three times a day, for its tonic and stimulant effect on the heart. The diet was first liquid; bouillon, milk, etc., in small quantities at frequent intervals, then gradually increased in variety, omitting sweets and fats. Improvement began immediately. Tetany disappeared, morphine was stopped in ten days, the nausea and vomiting were relieved, appetite reappeared, and she gained forty-three pounds since treatment was instituted, weighing now 113 pounds. She says she is better than she has been for years.

*513 East Grace street.*

